# Netting and pan traps fail to identify the pollinator guild of an agricultural crop

**DOI:** 10.1038/s41598-020-70518-9

**Published:** 2020-08-14

**Authors:** K. J. Boyer, F. P. Fragoso, M. E. Dieterich Mabin, J. Brunet

**Affiliations:** 1 Vegetable Crop Research Unit, United States Department of Agriculture, Agricultural Research Service, Madison, WI USA; 2 Oak Ridge Institute for Science and Education, Agricultural Research Service Research Participation Program, Madison, WI USA

**Keywords:** Agroecology, Conservation biology

## Abstract

Pollinator decline is expected to cause significant reductions in food production and plant reproduction. Quantifying the impact of pollinator decline on food production requires survey methods that can identify insect and bee species responsible for pollination of specific crops. To address this issue, we compared the effectiveness of two survey methods, netting and pan traps, at capturing the pollinators of alfalfa, *Medicago sativa.* Alfalfa is a major component of forage for cows and an important ingredient in chicken feed. We also examined bee species richness and diversity with these two survey methods, and compared these measures among three different colors of pan traps. Netting was more effective at capturing known pollinators of alfalfa, especially those belonging to the *Bombus* and *Apis* genera. Pan traps captured a higher bee diversity relative to netting and, like previous studies, each survey method and each trap color was more efficient at capturing certain bee genera. However, without a priori knowledge of pollinators, neither survey method could identify which of the bee species captured could pollinate alfalfa. We therefore recommend direct observations when the goal of a study is to identify pollinators or link pollinator decline to food production.

## Introduction

Animal pollination benefits approximately 87% of flowering plants worldwide^1^ and 35% of crops grown for human consumption^2^. Recent trends in global agricultural production have revealed an increasing reliance on animal pollination with more acreages being planted to pollinator-dependent crops, especially in developing countries^3,4^. For pollinator-dependent crops like pumpkin, coffee and watermelon, yield is often improved when a rich and diverse assemblage of wild pollinators is present^5–7^. A pollination management strategy that combines single species managed pollination with a diversity of wild pollinators has been recommended for insect-pollinated crops^8^. Because wild pollinators can contribute significantly to crop production and because globally, pollination services have been estimated at between $237 and $577 billion USD^9^, conserving wild pollinators has become a priority.

Survey methods that effectively quantify bee abundance and diversity are essential to monitor their demographic changes and allow the development of conservation methods. Direct pollinator observations and indirect methods have been used to quantify bee richness and diversity^10,11^. Researchers often use indirect survey methods such as pan traps and walking transects with a net^10,12^ because they are easier to use and require less manpower relative to direct pollinator observations^11^. When comparing indirect methods, netting is more labor intensive and subject to collector biases because it takes practice to reliably sample the majority of species present in an area, as many small bee species can be difficult to notice and catch in a net. Even experienced collectors may have difficulty seeing the smallest bees. In contrast, pan traps are easy to use, lack collector bias and can capture a greater richness and abundance of pollinator species^11,13–15^. However, pan traps may also be subject to biases because larger insects can escape drowning in them and they don’t attract all bee species. Standard protocols using pan traps are also easier to implement relative to protocols linked to sweep netting. These characteristics have led to the widespread popularity of pan traps as a survey method to quantify pollinator richness and diversity.

Pollinator decline is predicted to negatively impact food production and reproduction of many wild plant species^16–18^. In order to quantify the impact of pollinator decline on food production and plant reproduction, we need a survey method that can link bee abundance and diversity to pollination and capture the pollinator guild of a crop species or a plant community. Netting has been suggested as the most effective strategy to reach this goal because it catches insects while they are visiting flowers^19^. Pan traps, on the other hand, may catch many insects that do not play an active role in pollination of the local flora. Westphal et al.^11^ contrasted the performance of six sampling methods on a large scale and recommended pan traps as a reliable and cost-effective method to survey bee biodiversity while they suggest using transect walks with netting for studies focusing on plant/pollinator interactions. However, few studies have directly examined the efficiency of netting and pan traps at capturing the pollinator guilds of crop species or plant communities, an approach that directly links indirect survey methods to pollination.

The plant species *Medicago sativa*, commonly known as alfalfa or lucerne, is among the four most valuable field crops in the United States. It represents a major component of the forage used to feed cows and is an important ingredient in chicken feed. Flowers of this plant species are organized into racemes and a plant has many racemes per stem and stems per plant^20^. Many flowers may be open simultaneously within a raceme and among racemes on a plant. Insect pollination is required for seed production and pollinators must depress the keel of a flower in order to release the pollen and expose the stigma, in a process called tripping. Once a flower is tripped, the stigma and anthers remain pressed against the upper part of the flower, *i.e*. the flower remains open. The reliance of this plant species on pollinators for seed production makes it a great candidate to examine and compare the efficiency of survey methods in capturing pollinators of a plant species in order to link survey method to plant reproduction.

In this study, we examined and contrasted the efficiency of pan traps versus netting at capturing and identifying the pollinators of *Medicago sativa*. While pursuing this goal, we also compared the efficiency of pan traps versus netting at describing bee richness and diversity and bee composition in flowering patches of *M. sativa*. In addition, we examined the impact of the color of the pan traps on bee composition and bee diversity. We used and compared different measures of diversity and examined overall diversity and daily averages between survey methods and among pan trap colors. While it has been recommended to use transect walks with netting for studies focusing on plant pollinator interactions and to use pan traps as a reliable and cost-effective method to survey bee biodiversity, the current study directly examines which survey method best captures the pollinators of an agricultural crop.

## Results

### Bee abundance and sample coverage

We collected 691 individuals belonging to 43 species and to 11 genera. Overall, netting caught 526 individuals belonging to 29 species and pan traps 165 individuals from 35 species. Netting captured 76.1% of the individual bees collected in this study while pan traps only collected 23.9%. Per day, netting captured (Mean ± SEM) 52.4 ± 9.1 individual bees relative to 16.4 ± 3.4 bees for pan traps. Daily counts of each bee species captured via netting, pan traps, and each color of pan trap are presented in Supplementary Table S1 online. Per patch per day, 1.25 person hours were spent netting and 24 pan traps, eight of each color, were deployed for five hours. Of the 43 species, netting captured 67.4% of the bee species while pan traps caught 81.4% of the total species.

The three colors of pan traps caught similar number of bees each day (F_2,27_ = 1.35, P = 0.28). On a daily average, there were (mean ± s.e.m.) 5.3 ± 1.6 bees caught in the blue pan traps, 3.7 ± 0.9 bees in the yellow and 7.5 ± 2.0 in the white pan traps. Overall, the blue pan traps caught 53 bees, the yellow pan traps 37 and the white pan traps 75.

### Comparing diversity measures between survey methods and pan trap colors

#### Overall diversity measures

While the absolute values of the different diversity measures varied between the two survey methods and among the three colors of pan traps, their relative rankings remained the same (Table 1). Excluding *B. impatiens* increased bee diversity for netting but not for pan traps (Table 1). This finding is not surprising given that the majority of *B. impatiens* individuals were caught via netting (Table 2).

**Table 1:** Overall diversity measures for the two survey methods and the three pan trap colors when *B. impatiens* is included (all data) or excluded from the calculations.

	All data					Excluding *B. impatiens*				
	Netting	Trap	Blue	Yellow	White	Netting	Trap	Blue	Yellow	White
Species richness (S or q = 0)	29	35	20	14	21	28	34	19	13	20
Shannon–Wiener index (H)	1.41	2.80	2.60	2.08	2.48	2.23	2.75	2.52	1.98	2.40
Gini-Simpson index (D)	0.54	0.89	0.90	0.80	0.87	0.78	0.88	0.89	0.78	0.85
Shannon Diversity (q = 1)	4.10	16.44	13.46	8.00	11.94	9.30	15.64	12.43	7.24	11.02
Simpson Diversity (q = 2)	2.17	9.09	10.00	5.00	7.69	4.55	8.33	9.09	4.55	6.67

**Table 2:** Abundance (*n*) and proportion of the different bee genera captured by each survey method. Statistical differences between the two survey methods were determined using the log likelihood ratio test (G-test) with α = 0.05. Although presented in the Table for completeness, the genera *Augochlora, Agapostemom* and *Calliopsis* were not included in the statistical test.

Genus	Netting		Pan traps		Overall		
	*n*	Proportion within netting	*n*	Proportion within traps	Proportion caught by netting over total	Total abundance	P (goodness of fit test)
*Agapostemon*	0	–	1	0.01	–	1	N/A
*Andrena*	10	0.02	4	0.02	0.71	14	0.103
*Apis*	79	0.15	3	0.02	0.96	82	< 0.001
*Augochlorella*	10	0.02	35	0.21	0.22	45	0.192
*Augochlora*	1	0.00	2	0.01	0.33	3	N/A
*Bombus*	363	0.69	22	0.13	0.94	385	< 0.001
*Calliopsis*	0	–	3	0.02	–	3	N/A
*Halictus*	30	0.06	46	0.28	0.39	76	0.065
*Lasioglossum*	25	0.05	34	0.21	0.42	59	0.889
*Megachile*	7	0.01	4	0.02	0.64	11	0.363
*Peponapis*	1	0.00	11	0.07	0.08	12	0.002
Total	526		165			691	< 0.001

**Figure 1:**
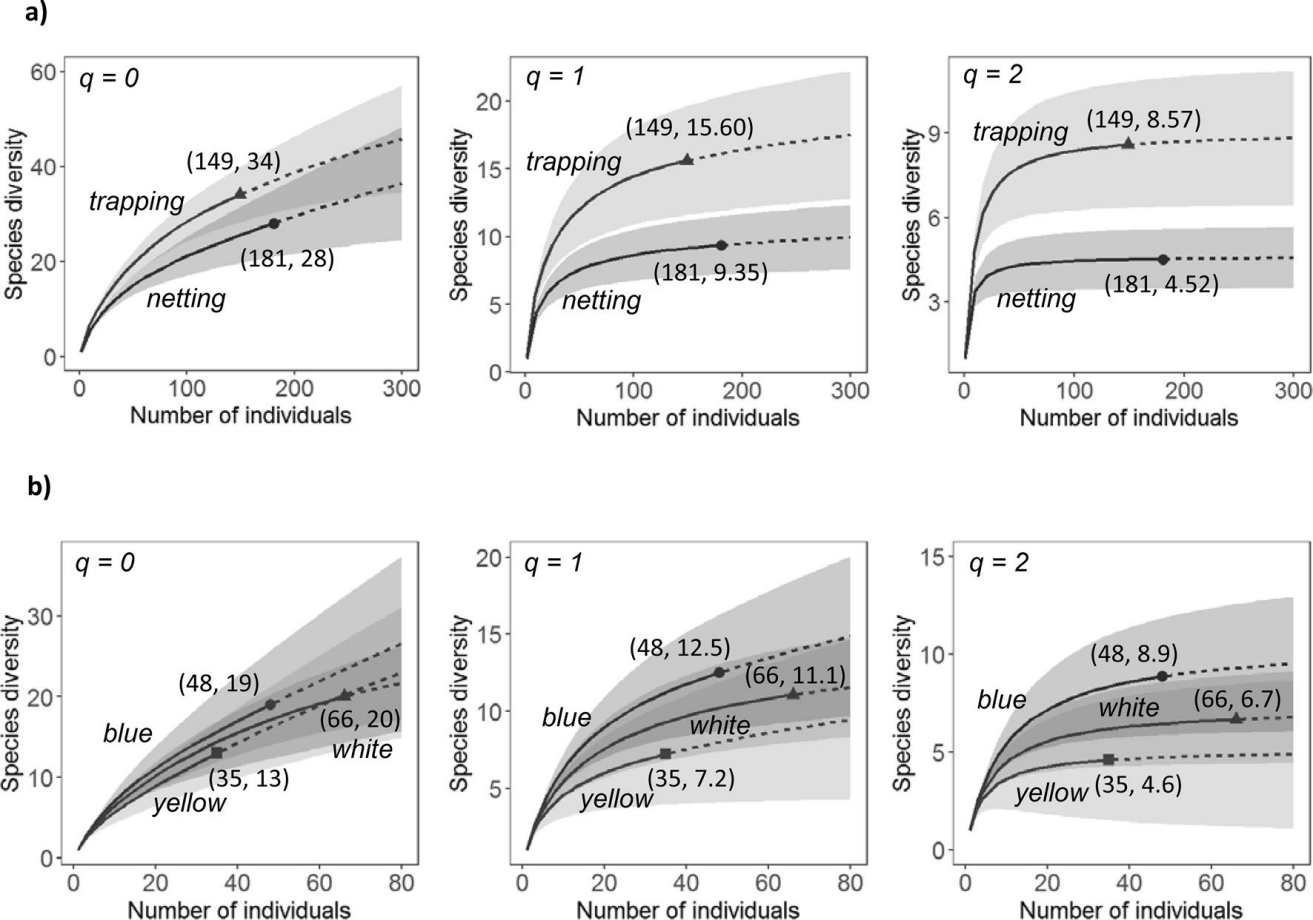
Sample-size-based rarefaction (solid line segment) and extrapolation (dashed line segment) sampling curves for bee diversity. Curves are presented between (**a**) survey methods and (**b**) pan trap colors. Bee diversity of order 0 (q = 0) is species richness, order 1 (q = 1) is Shannon diversity and order 2 (q = 2) is Simpson diversity. The gray-shaded areas represent the 95% confidence intervals and the solid circles, triangles and squares are the reference samples. Sample size and diversity measure are in parentheses. Diversity measures excluded *Bombus impatiens* individuals.

Based on rarefaction/extrapolation curves, pan traps caught a greater diversity of bees relative to netting and this was true for the number of bee species caught (species richness, q = 0), and for Shannon (q = 1) and Simpson (q = 2) diversity (Fig. 1a). The 95% confidence intervals overlapped for the number of species but not for Shannon or Simpson diversity (Fig. 1a). These curves permit comparisons of the number of species or diversity expected if the two methods, netting and trapping, had caught similar numbers of individual bees (Fig. 1a). Similar results were obtained when *B. impatiens* was included in the analyses (Supplementary Fig. S1 online). In addition, blue pan traps caught more bee species relative to white or yellow pan traps which caught similar species richness (Fig. 1b, q = 0). Finally, both Shannon (q = 1) and Simpson (q = 2) diversity were greater in blue relative to white pan traps and least for yellow pan traps (Fig. 1b, q = 1 and 2). When comparing pan traps of different colors, the 95% confidence intervals overlapped for all three diversity measures (Fig. 1b).

#### Daily diversity measures

##### Species richness

The two survey methods collected similar average number of bee species each day (F_1,18_ = 0.61, *P* = 0.44), with (Mean ± SEM) 6.8 ± 1.7 bee species captured via netting and 8.7 ± 1.5 via pan traps. Moreover, the average number of bee species caught each day did not differ among pan traps of different colors (F_2,27_ = 1.45, *P* = 0.25). Blue pan traps captured a daily average of 3.8 ± 1.0 bee species relative to 2.4 ± 0.6 for yellow and 4.7 ± 1.5 for white pan traps. Similar results were obtained when *B. impatiens* was removed prior to the calculation of the diversity measures. Without *B. impatiens*, 5.8 ± 1.8 bee species were captured via netting and 7.9 ± 1.6 via pan traps (F_1,18_ = 0.76, *p* = 0.39). Pan traps of different colors caught similar average number of bee species each day (F_2,27_ = 1.09, *P* = 0.35), with a daily average of 3.5 ± 1.0 bee species in blue, 2.2 ± 0.6 in yellow and 4.1 ± 1.1 in white pan traps.

##### Shannon (q = 1) and Simpson (q = 2) diversity

Statistical analyses based on daily estimates of the Shannon–Wiener (H) index or the Gini-Simpson index (D) provided similar results to the analyses based on daily estimates of Shannon (q = 1) or Simpson (q = 2) diversity. We therefore only present the results based on Shannon and Simpson diversity. Pan traps captured greater bee diversity relative to netting and this was true for both Shannon diversity (F_1,16_ = 10.49, *P* = 0.005) and Simpson diversity (F_1,16_ = 15.47, *P* = 0.001) (Table 3). When *B. impatiens* individuals were excluded, Shannon (F_1,16_ = 2.67, *P* = 0.122) and Simpson (F_1,14_ = 1.80, *P* = 0.201) diversity no longer differed statistically between pan traps and netting. This resulted from an increase in bee diversity for netting and a slight decrease for pan traps (Table 3). Neither Shannon (F_2,24_ = 1.48, *P* = 0.25) or Simpson diversity (F_2,22_ = 0.95, *P* = 0.40) differed among pan traps of different colors when diversity measures included *B. impatiens*. Similar results were obtained when *B. impatiens* individuals were excluded from the diversity calculations with (F_2,24_ = 1.21, *P* = 0.31) for Shannon diversity and (F_2,21_ = 1.01, *P* = 0.38) for Simpson diversity (Table 3).

**Table 3:** Daily averages (Mean ± SEM) for diversity measures for the two survey methods (n = 20) and the three colors of pan traps (n = 30). The diversity measures are described in the text and were calculated using all data or excluding *B. impatiens* individuals. Different letters indicate statistical differences between treatments (survey methods or pan trap colors) based on analysis of variance with α = 0.05 and multiple means comparison test for color of pan traps. Statistical results are presented in the text.

	Net	Trap	Blue	Yellow	White
**All data**					
Shannon–Wiener (H)	0.90 ± 0.23^a^	1.91 ± 0.13 ^b^	1.20 ± 0.20^a^	0.74 ± 0.20 ^a^	1.26 ± 0.25 ^a^
Gini–Simpson (D)	0.39 ± 0.09 ^a^	0.80 ± 0.02^b^	0.73 ± 0.05^a^	0.55 ± 0.09 ^a^	0.59 ± 0.11 ^a^
Shannon diversity (q = 1)	3.04 ± 0.62 ^a^	7.27 ± 1.06 ^b^	3.97 ± 0.78^a^	2.56 ± 0.61^a^	4.43 ± 0.84^a^
Simpson diversity (q = 2)	2.07 ± 0.29^a^	5.68 ± 0.72 ^b^	3.97 ± 0.59^a^	2.68 ± 0.57^a^	3.85 ± 0.64^a^
**Excluding ***B. impatiens*					
Shannon–Wiener (H)	1.15 ± 0.25^a^	1.77 ± 0.14^a^	1.08 ± 0.23^a^	0.64 ± 0.20^a^	1.14 ± 0.24^a^
Gini–Simpson (D)	0.73 ± 0.06^a^	0.77 ± 0.02^a^	0.67 ± 0.09^a^	0.48 ± 0.11^a^	0.67 ± 0.09^a^
Shannon diversity (q = 1)	4.07 ± 0.89^a^	6.45 ± 1.0^a^	3.67 ± 0.77^a^	2.36 ± 0.60^a^	3.95 ± 0.79^a^
Simpson diversity (q = 2)	3.50 ± 0.53^a^	4.95 ± 0.68^a^	3.64 ± 0.59^a^	2.46 ± 0.58^a^	3.74 ± 0.60^a^

### Bee composition

The two survey methods differentially captured bee genera (Table 2). For example, bumble bees (*Bombus)* represented 69%, and *Apis*, the honey bee, 15% of the total bees collected via netting. In contrast, *Halictus* bees represented 28% of the total bees collected by pan traps, followed by bees in the genera *Augochlorella* (21%) and *Lasioglossum* (21%) (Table 2). Netting captured 94% of the bumble bees caught in this study, 96% of honey bees and 71% of the *Andrena* bees. In contrast, pan traps caught 92% of *Peponapis* bees, 78% of *Augochlorella* bees and 61% of *Halictus* bees (Table 2).

**Figure 2:**
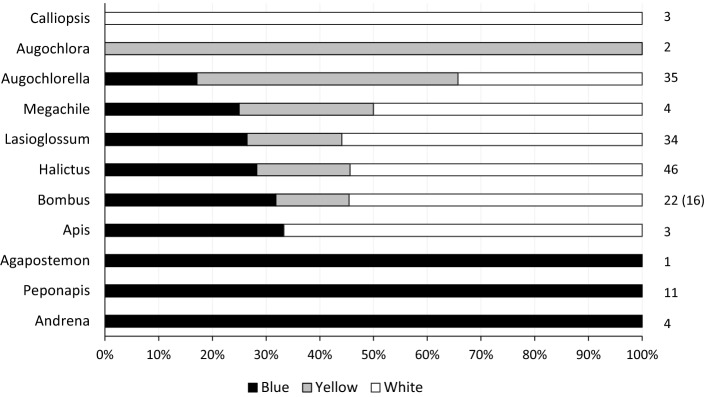
Proportion of individual bees from each bee genus captured by each color pan trap. The number at the end of a row represents the total number of bees of a given genus caught by pan traps. For the genus *Bombus*, the number in parenthesis indicates the number of individuals when the species *B. impatiens* is excluded.

The traps of distinct colors also caught different types of bees (Fig. 2; Table 4). For example, bees in the genera *Andrena, Peponapis* and *Agapostemon* were only caught in blue pan traps in contrast to bees of the genus *Augochlora* which were only captured in yellow pan traps and bees of the genus *Calliopsis* in the white pan traps (Fig. 2; Table 4). In addition, bumble bees, honey bees and bees in the genera *Halictus* and *Lasioglossum* were captured in greater number in white pan traps (Fig. 2; Table 4). Many genera were captured in traps of all three colors (Fig. 2; Table 4).

**Table 4:** The bee species sampled in alfalfa patches using netting or colored pan traps.

Family/Species	Net	Trap	Blue	Yellow	White	Total
**Andrenidae**						
* Andrena crataegi* Robertson, 1893	0	3	3	0	0	3
* Andrena miranda* Smith, 1879	10	1	1	0	0	11
* Calliopsis andreniformis* Smith, 1853	0	3	0	0	3	3
**Apidae**						
* Apis mellifera* Linnaeus, 1761	79	3	1	0	2	82
* Bombus affinis* Cresson, 1863	5	0	0	0	0	5
* Bombus auricomus* Robertson, 1903	2	2	0	1	1	4
* Bombus bimaculatus* Cresson, 1863	0	1	1	0	0	1
* Bombus citrinus* Smith, 1854	0	1	0	0	1	1
* Bombus griseocollis* De Geer, 1773	1	0	0	0	0	1
* Bombus impatiens* Cresson, 1863	345	16	5	2	9	361
* Bombus rufocinctus* Cresson, 1863	3	1	0	0	1	4
* Bombus vagans* Smith, 1854	7	1	1	0	0	8
* Peponapis pruinosa* Say, 1874	1	11	11	0	0	12
**Halictidae**						
* Agapostemon splendens* Lepeletier, 1841	0	1	1	0	0	1
* Augochlora pura* Say, 1837	1	2	0	2	0	3
* Augochlorella aurata* Smith, 1873	1	30	5	14	11	31
* Augochlorella gratiosa* Smith, 1853	0	1	1	0	0	1
* Augochlorella persimilis* Viereck, 1910	9	4	0	3	1	13
* Halictus confusus* Smith, 1853	24	36	8	7	21	60
* Halictus ligatus* Say, 1873	0	8	4	1	3	8
* Halictus parallelus* Say, 1837	1	0	0	0	0	1
* Halictus rubicundus* Christ, 1791	5	2	1	0	1	7
* Lasioglossum albipenne* Robertson, 1,890	3	5	1	1	3	8
* Lasioglossum coriaceum* Smith, 1853	1	1	0	1	0	2
* Lasioglossum foxii* Robertson 1895	1	1	1	0	0	2
* Lasioglossum gotham* Gibbs, 2011	0	1	0	0	1	1
* Lasioglossum heterognathum* Mitchell, 1960	0	3	0	1	2	3
* Lasioglossum hitchensi* Gibbs, 2012	0	2	0	0	2	2
* Lasioglossum illinoense* Robertson 1892	1	0	0	0	0	1
* Lasioglossum imitatum* Smith, 1853	8	3	0	1	2	11
* Lasioglossum laevissimum* Smith, 1853	1	3	3	0	0	4
* Lasioglossum macoupinense* Robertson,	1	0	0	0	0	11
* Lasioglossum pilosum* Smith, 1853	0	1	1	0	0	1
* Lasioglossum platyparium* Robertson, 1895	0	3	2	0	1	3
* Lasioglossum pruinosum* Robertson, 1892	1	1	0	0	1	2
* Lasioglossum sagax* Sandhouse, 1924	0	1	0	0	1	1
* Lasioglossum smilacinae,* Robertson, 1897	1	0	0	0	0	1
* Lasioglossum tegulare* Robertson, 1,890	1	0	0	0	0	1
* Lasioglossum versans* Lovell, 1905	4	1	0	1	0	5
* Lasioglossum versatum* Robertson, 1902	2	8	1	1	6	10
**Megachilidae**						
* Megachile brevis* Smith, 1853	0	1	1	0	0	1
* Megachile concinna* Smith, 1879	6	3	0	1	2	9
* Megachile mendica* Cresson, 1878	1	0	0	0	0	1

We observed notable differences in the bee species collected by the two survey methods. Eight of the 43 bee species captured in this study (18.6%) were only collected using the netting method, while 14 species (32.6%) were only found in pan traps (Table 4). The remaining 21 species (48.8%) could be caught by either method although, for some of these bee species, one of the two methods captured more individuals (Table 4).

## Discussion

In order to quantify the impact of pollinator decline on food production and plant reproduction, we need a survey method that adequately captures the pollinators of plant species. Pan traps and netting can capture different bee composition^12,19^. In general, species in the genera *Apis, Bombus,* and *Megachile* are poorly represented in pan traps, while species in the family Halictidae are commonly caught. In the current study, we found a preponderance of bumble bees, honey bees and bees from the genus *Andrena* in the netting samples. In contrast, pan traps were better at capturing bees from the genera *Halictus* and *Augochlorella*. These results indicate that using both survey methods provides a better representation of the visitors to patches of alfalfa flowers.

Previous direct observations of insects visiting and tripping alfalfa flowers, an indication of an alfalfa pollinator, have identified bumble bees and some *Halictus* and *Andrena* bees as common wild pollinators in the study area^21^. These two wild solitary bee genera have been identified as important pollinators of alfalfa in other parts of the world, together with additional solitary bee genera that include *Megachil*e, *Eutricharaea*, and *Anthophora* amongst others^22,23^. These studies all identified pollinators of alfalfa via direct pollinator observations and some quantified their efficiency by measuring their tripping rate^20,24^. In alfalfa seed-production fields in the western United States, managed pollinators are brought to the fields. The alfalfa leafcutting bee, *Megachile rotundata*, is used in the Pacific Northwest with increasing usage in California. Honey bees, *Apis mellifera*, are more commonly used in California. Alkali bees, *Nomia melanderi,* are used in part of the Pacific Northwest but their use is restricted to specific regions due to their stringent environmental requirements. Bumble bees, *Bombus impatiens* in North America, are commonly used in greenhouses and in seed increase associated with alfalfa breeding. These managed bees have different tripping rates, a measure of pollinator efficiency in alfalfa, with *Nomia*, *Megachile* and *Bombus* species all having higher tripping rates than honey bees^21,24^.

The bee composition in our two survey methods suggests that the efficiency of netting relative to pan traps in catching pollinators of a plant species or community will vary with the bee genera. Netting provided a better representation of the bumble bees (*Bombus*), honey bees (*Apis*) and *Andrena* species in our study area but it tended to underestimate the abundance of *Halictus* (Table 2). We could establish from these findings that netting was a better method to capture pollinators of alfalfa. However, this is only due to our prior knowledge of the pollinators of alfalfa in the area. Neither netting or pan traps provide any indication of which of the bee genera captured in the survey truly pollinate alfalfa. Looking for pollen on a bee’s body and, when possible, identifying the type of pollen could help confirm or eliminate some bees as pollinators. But identifying pollen is time consuming and examining pollen is more likely to work for bees caught via netting than pan traps. Netting caught *Lasioglossum* bees which have been reported^25^ visiting alfalfa flowers for pollen in Utah, and have been previously caught via netting in Canada^26^. Netting also caught various bee genera that have not been reported as alfalfa pollinators in this area and, to our knowledge, in other parts of the world. Both netting and pan traps caught bees from the genera *Augochlorella*, *Augochlora* and *Peponapis* and, in addition, pan traps also caught bees from the genera *Agapostemon* and *Calliopsis*. One *Calliopsis* bee species has previously been observed on alfalfa in Nebraska^27^and bees of the genus *Agapostemon* have been captured via netting in Canada^26^. Both survey methods caught bees very unlikely to be involved in alfalfa pollination. One concern with the use of indirect survey methods such as pan traps and sweep netting is that they can catch a variety of insects that are simply flying around the area or may be visiting a plant but are stealing nectar or eating pollen without contributing to its pollination. Results of this study indicate that these concerns are substantiated.

Pan traps caught a greater diversity of bees relative to netting. This statement was supported by the comparisons of overall bee diversity using the three integrated rarefaction/extrapolation curves, one based on species richness (q = 0), a second on Shannon diversity (q = 1) and a third on Simpson diversity (q = 2)^28^_._ Daily diversity averages also differed significantly between the two survey methods when all data were considered but not when *B. impatiens* individuals were removed prior to quantifying diversity measures. The lack of statistical significance in daily diversity measures in the absence of *B. impatiens* resulted from an increase in diversity under netting, which caught the great majority of *B. impatiens* individuals, rendering its diversity closer to the one obtained from pan traps. These results support the use of overall diversity measures and associated statistical tests when comparing among treatment methods or communities.

The color of a pan trap can influence which insects they attract^10,12,13,15,29^. In general, blue, white and yellow pan traps catch a wide range of bee species, and outperform other colors such as red, green, pink, orange and turquoise^12,30,31^. The effectiveness of different pan trap colors can vary with habitat^15^ and species composition^19^. In this study, the integrated rarefaction/extrapolation curves^28^ comparing overall diversity suggest that blue pan traps caught more bee species (q = 0) than white or yellow traps and that both Shannon (q = 1) and Simpson (q = 2) diversity were greater in blue than white and greater in white relative to yellow pan traps. However, based on average daily diversity, the color of the pan trap did not affect bee richness or bee diversity. The different colors of pan traps did, however, demonstrate biases in the genera of bees they captured^10,12,13,15,29,32^. For the bee genera where at least 10 individuals were caught in pan traps, bees of the genus *Augochlorella* were preferentially caught in yellow traps while bees from the genera *Bombus*, *Halictus* and *Lasioglossum* were caught more frequently in white traps. The fact that white traps caught slightly more bumble bee individuals relative to the blue traps in this study suggests that there might be some UV reflectance in the white traps we used. The results of this study support the recommendation of using traps of different colors to adequately represent bee abundance and bee diversity^11^. However, while some studies have reported a relationship between bee diversity and crop yield^6,7^, such an association is only expected when the increase in bee diversity reflects an increase in pollinator diversity and when the crop or plant community is otherwise pollen-limited^33^and increasing pollinator diversity overcomes pollen limitation.

Despite the small scale of this study, the results illustrate how each survey method and pan trap color tend to capture distinct bee genera and differentially affect bee diversity and, in this respect, results support the conclusions of previous studies^10–13,19^. Survey methods that effectively quantify composition and diversity are essential to monitor bee demographic changes and allow the development of conservation methods. While using pan traps alone may be easier and more economical, each method has its own biases and the two methods complement each other. We recommend the use of both survey methods to obtain a good representation of bee composition, bee richness and bee diversity. The main goal of this study, however, was not to examine the ability of these two survey methods and of pan traps of distinct colors to capture bee composition, bee richness and bee diversity. It was not either to determine whether insects caught via netting or in the pan traps shared traits that may increase the chance of them being alfalfa pollinators. The main goal of this study was to determine whether these two survey methods could identify the pollinators of a crop species, in this case, alfalfa. Identifying pollinators is a prerequisite for a survey method to link bee diversity or pollinator decline to food production^16–18^. Results of this study indicate that, without a priori knowledge, neither survey method could identify pollinators of alfalfa. Both netting and pan traps caught many of the known pollinators of alfalfa, although netting caught many more honey bees and bumble bees relative to pan traps. Both methods also captured other bees that are not known to pollinate alfalfa. Because bee abundance alone is not a good indicator of pollination and its efficiency^34^, without a priori knowledge, these methods provide little information about which bees are most likely to be pollinators. We therefore discourage the use of pan traps and netting and recommend the use of direct pollinator observation methods when the main objective of a study is to identify pollinators of a plant species or link pollinator decline to food production.

## Methods

### Plant species

*Medicago sativa* is a perennial plant and alfalfa cultivars are mostly tetraploid and self-compatible. Bees are required for seed production and alfalfa seed production fields tend to have high outcrossing rates^35^. The majority of selfing in alfalfa seed production fields may result from geitonogamous selfing, a mode of selfing where pollinators move pollen among flowers on a plant^35^.

Different managed pollinators are used in alfalfa seed production^36^. The alfalfa leafcutting bee, *Megachile rotundata*, is the major pollinator of alfalfa in the Pacific Northwest and is often used together with the honey bee, *Apis mellifera,* in California. The alkali bee, *Nomia melanderi*, has strict habitat requirements which limits its use to the Walla Walla region in the Pacific Northwest. Various wild pollinators, including bumble bees (*Bombus sp.)* and mining bees (*Andrena sp.)* also visit alfalfa flowers^21,25,27^.

### Experimental set up

This study took place at the West Madison Agricultural Research Station, an experimental farm situated in a suburban-agricultural landscape in Madison, Wisconsin. Two sites were selected for this study. A later trimming date for alfalfa during late spring-early summer delayed peak alfalfa flowering at one of the sites by about two weeks. Using these two sites extended the period over which insects visiting alfalfa flowers could be collected. Data were gathered over five days at each site during the summer of 2018. At the first site, traps were set up and netting took place between July 30 and August 3 while at the second site, data were collected on August 22–23 and 27–29, interrupted by a period of rain.

Each site consisted of a center patch and four peripheral patches of alfalfa that were set up for a distinct experiment. At each site, we set up pan traps and did the netting in transects as detailed below in three 82.8m^2^ peripheral patches. Grass was growing between the patches and mowed periodically. The experiment was surrounded by alfalfa hay fields on three sides, and a vehicle path on the fourth side. At each site, two hives of *Bombus impatiens* were located at the edge of the center patch.

### Survey methods

#### Pan traps

**Figure 3:**
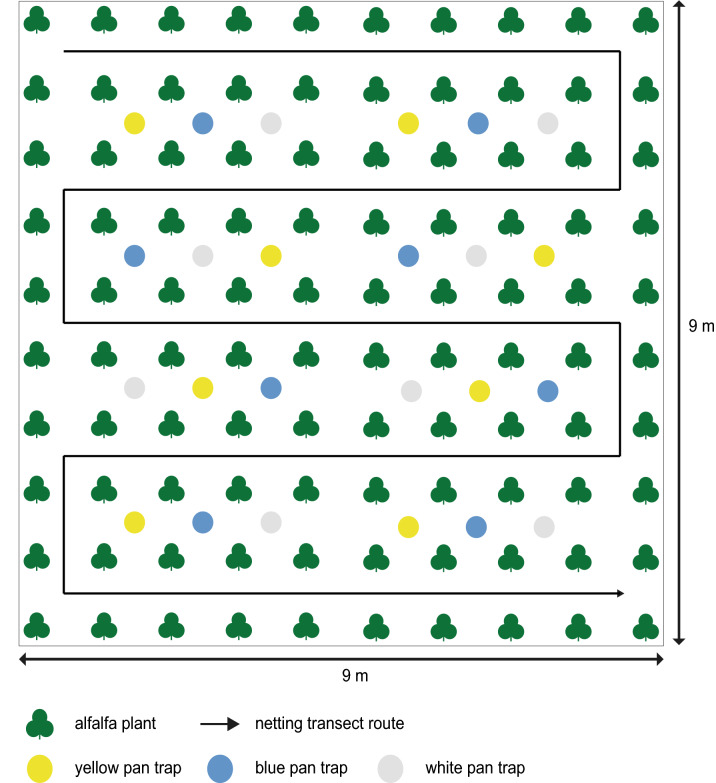
Arrangement of pan traps within an alfalfa patch. The green represents alfalfa plants while the white, blue and yellow dots illustrate the location of the pan traps of respective colors. The black line illustrates the transect lines used during netting.

White, yellow, and blue pan traps were used to passively collect insects visiting patches of flowering alfalfa. These colors were selected because they have been widely used^10,29,30,37^, and represent a wide range of wavelengths on the visible spectrum. Colored plastic bowls with a diameter of 15.5 cm and a depth of 4 cm were filled to a depth of approximately 2.5 cm with water containing a few drops of Seventh Generation™ unscented soap. Eight pan traps of each color were set up in each of the three alfalfa patches, for a total of 24 pan traps per patch and 72 pan traps at each site (Fig. 3). Pan traps were arranged in an alternating pattern along 4 transects in each alfalfa patch, such that no two adjacent pan traps were of the same color (Fig. 3). Pan traps were set up each day of observation by 9:00 AM and were removed at 2:00 PM. Insects collected in pan traps were stored in plastic vials in 95% ethanol until they were pinned and identified in the laboratory.

#### Netting

In this standardized transect walk, a series of five linear east–west transects were set up in each patch (Fig. 3). Transects were walked for a total of 15 min every hour, and flower-visiting insects were collected as they were spotted on flowers. Three observers simultaneously walked the transects, one in each patch. A total of six observers participated in the surveys and observers rotated among patches in order to randomize data collection. At the end of the 15-min period we identified some of the bees and kept in a plastic vial the bees that could not be identified in the field. The vials were placed on ice and stored in a freezer upon return to the laboratory until they could be pinned and identified.

### Bee identification and sample coverage

Bumble bee species were identified using the USDA Forest Service Guide to Bumblebees of the Eastern United States. All other bee species were identified using taxonomic keys. All bees were identified to the species level. Overall, 2.67% of the samples (19/711) were too damaged for species identification and were excluded from analyses.

Sample coverage of each survey method was tabulated as the proportion of species captured by a survey method, *i.e.* the number of species captured by a survey method relative to the total number of species captured in the experiment (both methods combined).

### Diversity measures

Different diversity measures were tabulated. While species richness represents the number of species, all other diversity measures include both species number and species abundance in their calculations. The Shannon–Wiener diversity index is $${\text{H}} = - \sum\nolimits_{{{\text{i}} = 1}}^{{\text{S}}} {{\text{p}}_{{\text{i}}} \ln \;{\text{p}}_{{\text{i}}} }$$**,** where *pi* is the proportion of species *i, S* is the number of species and ln is the natural logarithm. This index weights each species based on its abundance alone and does not favor rare or common species. The Gini-Simpson diversity index, $${\text{D}} = 1 - \sum\nolimits_{{{\text{i}} = 1}}^{{\text{S}}} {{\text{p}}_{{\text{i}}}^{2} }$$, is a dominance index that gives more weight to common or dominant species. The Shannon–Wiener index and the Gini-Simpson index were tabulated using the ‘vegan’ package^38^ in R version 3.6.1^39^. We also calculated diversity measures based on Hill numbers, of order 0 (q = 0) equivalent to species richness, order 1 (q = 1) or Shannon diversity and order 2 (q = 2) or Simpson diversity, where *q* reflects how sensitive the measure is to relative abundance^40^. Shannon diversity is equivalent to the exponential of the Shannon–Wiener index and Simpson diversity to the inverse of the Simpson concentration, $${\raise0.7ex\hbox{$1$} \!\mathord{\left/ {\vphantom {1 {\sum\nolimits_{{{\text{i}} = 1}}^{{\text{S}}} {{\text{p}}_{{\text{i}}}^{2} } }}}\right.\kern-\nulldelimiterspace} \!\lower0.7ex\hbox{${\sum\nolimits_{{{\text{i}} = 1}}^{{\text{S}}} {{\text{p}}_{{\text{i}}}^{2} } }$}}$$^40^. These diversity indices were tabulated over the entire study and as daily averages. To determine the impact of the bumble bee hives on bee diversity, we calculated the various diversity measures including or excluding *B. impatiens* individuals.

### Survey methods, pan trap colors and bee diversity

Data from the two sites were combined prior to calculating diversity measures in order to include all bees collected over the alfalfa flowering period. To compare overall bee diversity between survey methods and among pan traps of different colors, we used three integrated rarefaction/extrapolation curves based on species richness (q = 0), Shannon diversity (q = 1) and Simpson diversity (q = 2)^40^. These curves were created using the iNEXT package^41^ in R version 3.6.1^39^.

Average daily diversity measures were tabulated for all diversity measures and compared between the two survey methods (n = 20) and among the pan traps of different colors (n = 30) using one-way analyses of variance (ANOVA) with α = 0.05. Day was the replicate in the ANOVA models and analyses were done on diversity measures calculated including or excluding *B. impatiens* individuals. Plots of the models’ residuals versus predicted values were examined to determine whether the model was a good fit to the data.

### Survey methods and bee genera

We used a G-goodness of fit test, also known as the likelihood or log-likelihood ratio test, with α = 0.05, to determine if the two survey methods resulted in the collection of similar abundances of each bee genus or if one method was better at collecting some genera over others. The G-goodness of fit test examined the null hypothesis that the same proportion of individuals were caught by these two survey methods. Species were grouped into genera for this test in order to obtain the minimum sample size of five individuals expected per category. Because we did not catch sufficient individuals of the genera *Agapostemon*, *Augochlora* and *Calliopsis* to meet the assumptions of the test, these genera were removed from this analysis. These tests were performed in R version 3.6.1^39^ using the package ‘DescTools’^42^.

## Supplementary information

Supplementary file1.

## Data Availability

All data from this study are included in this publication and its Supplementary Material.
