# Supplementary material for: Netting and pan traps fail to identify the pollinator guild of an agricultural crop
Source: Sci Rep. 2020 Aug 14;10:13819. doi: 10.1038/s41598-020-70518-9 (PMC7427967; doi:10.1038/s41598-020-70518-9)
Supplement: Supplementary file 1 — Supplementary file1. [file 41598_2020_70518_MOESM1_ESM.pdf]

Netting and pan traps fail to identify the pollinator guild of an agricultural crop

K. J. Boyer[1], F. P. Fragoso[2], M. E. Dieterich Mabin[1] and J. Brunet[1*]

[1] United States Department of Agriculture, Agricultural Research Service, Vegetable Crop Research Unit, Madison, Wisconsin

[2] Oak Ridge Institute for Science and Education – Agricultural Research Service Research Participation Program, Madison, Wisconsin

***Correspondence to Johanne Brunet (johanne.brunet@usda.gov)

Supplementary Figure S1. Sample-size-based rarefaction (solid line segment) and extrapolation (dashed line segment) sampling curves for bee diversity including *Bombus impatiens*. Curves are presented between a) survey methods and b) pan trap colors. Bee diversity of order 0 (q= 0) is species richness, order 1 (q= 1) is Shannon diversity and order 2 (q= 2) is Simpson diversity. The gray-shaded areas represent the 95% confidence intervals and the solid circles, triangles and squares are the reference samples. Sample size and diversity measure are in parentheses. Diversity measures included all sampled individuals.

A

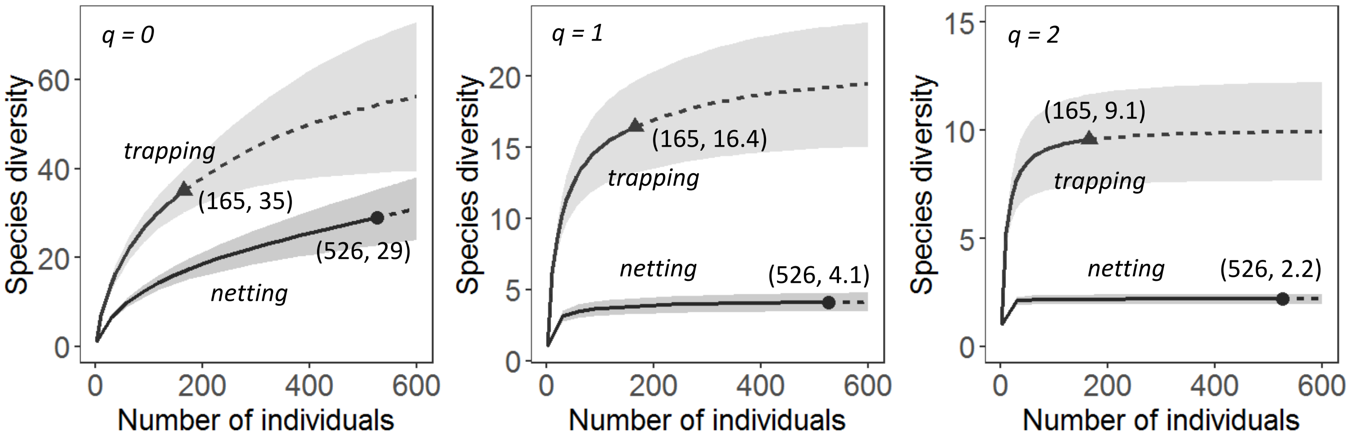

B

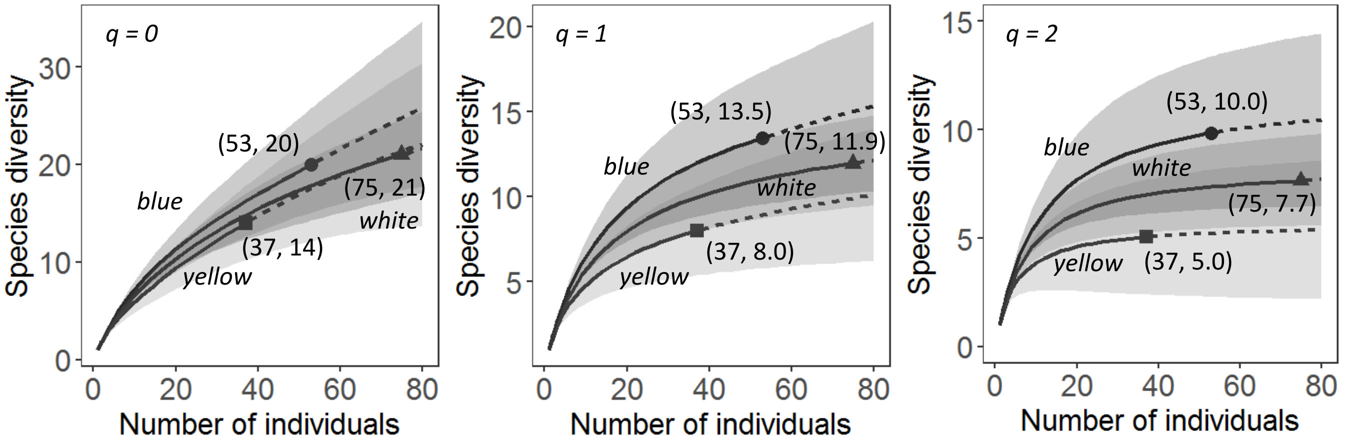

Supplementary Table S1. Daily counts of each bee species captured via netting, pan traps, and each color of pan trap. Total is 689 individuals, as the date was not known for two *Bombus* (one *Bombus affinis* and one *Bombus auricomis*) caught by netting.

Date	Species	netting	trap	blue	white	yellow
7/30/2018	Andrena miranda	4	1	1		
7/30/2018	Apis mellifera	8	0			
7/30/2018	Augochlora pura	1	0			
7/30/2018	Augochlorella aurata	1	0			
7/30/2018	Augochlorella gratiosa		1	1		
7/30/2018	Augochlorella persimilis	2	0			
7/30/2018	Bombus affinis	2	0			
7/30/2018	Bombus impatiens	37	1		1	
7/30/2018	Bombus vagans	2	0			
7/30/2018	Halictus confusus	8	0			
7/30/2018	Halictus rubicundus	3	0			
7/30/2018	Lasioglossum albipenne	2	0			
7/30/2018	Lasioglossum platyparium		2	2		
7/30/2018	Megachile concinna		1			1
7/30/2018	Megachile mendica	1	0			
7/31/2018	Andrena miranda	1	0			
7/31/2018	Apis mellifera	20	1		1	
7/31/2018	Augochlorella aurata		6		2	4
7/31/2018	Augochlorella persimilis	3	0			
7/31/2018	Bombus affinis	1	0			
7/31/2018	Bombus griseocollis	1	0			
7/31/2018	Bombus impatiens	49	0			
7/31/2018	Bombus rufocinctus	2	0			
7/31/2018	Bombus vagans	2	0			
7/31/2018	Halictus confusus	11	10	1	6	3
7/31/2018	Halictus ligatus		4	2	2	
7/31/2018	Halictus rubicundus	1	1		1	
7/31/2018	Lasioglossum albipenne	1	0			
7/31/2018	Lasioglossum foxii	1	0			
7/31/2018	Lasioglossum gotham		1		1	
7/31/2018	Lasioglossum heterognathum		1		1	
7/31/2018	Lasioglossum imitatum	2	1		1	
7/31/2018	Lasioglossum pruinosum	1	0			
7/31/2018	Lasioglossum smilacinae	1	0			
7/31/2018	Lasioglossum versans	1	0			
7/31/2018	Lasioglossum versatum		2		2	

7/31/2018	Megachile concinna	2	0			
7/31/2018	Peponapis pruinosa		1	1		
8/1/2018	Andrena miranda	1	0			
8/1/2018	Apis mellifera	15	0			
8/1/2018	Augochlorella persimilis	2	0			
8/1/2018	Bombus impatiens	30	2	1		1
8/1/2018	Bombus rufocinctus	1	0			
8/1/2018	Bombus vagans	3	0			
8/1/2018	Calliopsis andreniformis		1		1	
8/1/2018	Halictus confusus		4	2	2	
8/1/2018	Halictus rubicundus	1	0			
8/1/2018	Lasioglossum albipenne		1		1	
8/1/2018	Lasioglossum coriaceum	1	1			1
8/1/2018	Lasioglossum imitatum	2	0			
8/1/2018	Lasioglossum laevissimum		1	1		
8/1/2018	Lasioglossum tegulare	1	0			
8/1/2018	Lasioglossum versans	3	0			
8/1/2018	Lasioglossum versatum		1		1	
8/1/2018	Megachile concinna	1	0			
8/1/2018	Peponapis pruinosa		2	2		
8/2/2018	Andrena miranda	3	0			
8/2/2018	Apis mellifera	16	0			
8/2/2018	Augochlorella persimilis		1		1	
8/2/2018	Bombus auricomus		1			1
8/2/2018	Bombus impatiens	47	1		1	
8/2/2018	Calliopsis andreniformis		1		1	
8/2/2018	Halictus confusus	3	11	1	9	1
8/2/2018	Halictus ligatus		1		1	
8/2/2018	Lasioglossum hitchensi		1		1	
8/2/2018	Lasioglossum imitatum	1	1		1	
8/2/2018	Lasioglossum laevissimum		1	1		
8/2/2018	Lasioglossum sagax		1		1	
8/2/2018	Lasioglossum versatum		1		1	
8/2/2018	Peponapis pruinosa		1	1		
8/3/2018	Andrena crataegi		1	1		
8/3/2018	Andrena miranda	1	0			
8/3/2018	Apis mellifera	20	0			
8/3/2018	Augochlora pura		1			1
8/3/2018	Augochlorella aurata		4	1	2	1
8/3/2018	Augochlorella persimilis	1	0			
8/3/2018	Bombus affinis	1	0			
8/3/2018	Bombus auricomus	1	0			
8/3/2018	Bombus impatiens	48	2		2	

8/3/2018	Bombus rufocinctus		1		1	
8/3/2018	Bombus vagans		1	1		
8/3/2018	Calliopsis andreniformis		1		1	
8/3/2018	Halictus confusus	2	6	3	3	
8/3/2018	Halictus ligatus		1			1
8/3/2018	Halictus parallelus	1	0			
8/3/2018	Lasioglossum albipenne		2		1	1
8/3/2018	Lasioglossum heterognathum		2		1	1
8/3/2018	Lasioglossum hitchensi		1		1	
8/3/2018	Lasioglossum imitatum	3	0			
8/3/2018	Lasioglossum laevissimum		1	1		
8/3/2018	Lasioglossum pilosum		1	1		
8/3/2018	Lasioglossum platyparium		1		1	
8/3/2018	Lasioglossum pruinosum		1		1	
8/3/2018	Lasioglossum versans		1			1
8/3/2018	Lasioglossum versatum	1	2	1		1
8/3/2018	Peponapis pruinosa	1	1	1		
8/22/2018	Agapostemon splendens		1	1		
8/22/2018	Andrena crataegi		2	2		
8/22/2018	Augochlorella aurata		9	3	4	2
8/22/2018	Augochlorella persimilis		3			3
8/22/2018	Bombus bimaculatus		1	1		
8/22/2018	Bombus impatiens	34	4	3		1
8/22/2018	Halictus confusus		1			1
8/22/2018	Halictus ligatus		1	1		
8/22/2018	Lasioglossum albipenne		1	1		
8/22/2018	Lasioglossum foxii		1	1		
8/22/2018	Lasioglossum imitatum		1			1
8/22/2018	Megachile brevis		1	1		
8/22/2018	Peponapis pruinosa		3	3		
8/23/2018	Augochlorella aurata		5	1	2	2
8/23/2018	Bombus impatiens	41	2		2	
8/23/2018	Halictus confusus		3	1		2
8/23/2018	Halictus ligatus		1	1		
8/23/2018	Halictus rubicundus		1	1		
8/23/2018	Lasioglossum laevissimum	1	0			
8/23/2018	Lasioglossum macoupinense	1	0			
8/23/2018	Lasioglossum versatum	1	2		2	
8/23/2018	Megachile concinna	3	1		1	
8/23/2018	Peponapis pruinosa		3	3		
8/27/2018	Apis mellifera		1		1	
8/27/2018	Augochlora pura		1			1
8/27/2018	Augochlorella aurata		5		1	4

8/27/2018	Augochlorella persimilis	1	0			
8/27/2018	Bombus auricomus		1		1	
8/27/2018	Bombus citrinus		1		1	
8/27/2018	Bombus impatiens	31	2		2	
8/27/2018	Lasioglossum albipenne		1		1	
8/27/2018	Lasioglossum illinoense	1	0			
8/28/2018	Augochlorella aurata		1			1
8/28/2018	Bombus impatiens	19	0			
8/29/2018	Apis mellifera		1	1		
8/29/2018	Bombus impatiens	9	2	1	1	
8/29/2018	Halictus confusus		1		1	
8/29/2018	Megachile concinna		1		1	
		**524**	**165**	53	75	37
